# Mandibular Buccal Bifurcation Cyst (MBBC): A New Microsurgical and Guided Tissue Regeneration Approach—A Case Report

**DOI:** 10.1155/crid/6232691

**Published:** 2026-06-22

**Authors:** Casaña-Ruiz María Dolores, Boronat-Catalá Montserrat, Almiñana-Pastor Pedro, Velló-Ribes Ma Angeles, Catalá-Pizarro Montserrat

**Affiliations:** ^1^ Dentistry Department of Stomatology, Faculty of Medicine and Dentistry, University of Valencia, Valencia, Spain, uv.es; ^2^ Orthodontics Department, Faculty of Medicine and Dentistry, University of Valencia, Valencia, Spain, uv.es; ^3^ Periodontology Department, Faculty of Medicine and Dentistry, University of Valencia, Valencia, Spain, uv.es; ^4^ Paediatric Dentistry Department, Department of Stomatology, Faculty of Medicine and Dentistry, University of Valencia, Valencia, Spain, uv.es; ^5^ Paediatric Dentistry Department, Department of Stomatology, Faculty of Medicine and Dentistry, Valencia, Spain

**Keywords:** buccal bifurcation cyst, case report, MBBC, surgical approach GTR

## Abstract

**Background:**

Mandibular buccal bifurcation cyst (MBBC) is an inflammatory cyst that typically manifests during early childhood and is positioned buccal to the furcation area of the mandibular first molars. Clinically, it manifests as a buccal inclination of the crown without associated symptoms or dental vitality loss. The diagnosis is confirmed through a combination of clinical, radiological, surgical, and histological findings.

**Case Report:**

A case of a 9‐year‐old boy with MBBC is presented. Molar 4.6 exhibits a buccal inclination of the crown and a palpable, nonpainful hard swelling on the vestibular side of the tooth. Radiographic and cone‐beam computed tomography (CBCT) imaging revealed a lack of bone structure. A minimally invasive surgical procedure involving a micro‐osteotomy was performed, which enabled the en bloc excision of the cyst. A collagen membrane was used to reconstruct the tissues through complete regeneration, in accordance with the principles of guided tissue regeneration (GTR). After a 1‐year follow‐up period, complete restoration of the cortical bone and the lesion area is observed on a CBCT, with a completely asymptomatic tooth.

**Conclusions:**

A precise diagnosis of MBBC lesions is paramount for effective treatment, and this diagnosis must be made through clinical signs, radiographic imaging, and histology. Furthermore, it is imperative to ensure that surgical interventions are performed in a meticulous manner. A case is presented in which the lesion was treated by generating a bony microwindow to access the cyst and enucleate it in a single piece. Additionally, a collagen membrane was used to achieve complete regeneration of the lesion without interference from the soft tissues. The subsequent year of observation revealed a favorable evolution of the case, with complete regeneration of all lost tissues, including the buccal cortical.

## 1. Introduction

The buccal bifurcation cyst (BBC) was first described in 1983 by Stoneman and Worth. The World Health Organization (WHO) classifies the mandibular buccal bifurcation cyst (MBBC) as an inflammatory odontogenic cyst, arising from the pericoronal tissue [1]. Although some literature refers to this entity as a “paradental cyst,” the WHO recommends reserving that term exclusively for cysts associated with mandibular third molars [2].

BBCs account for approximately 5% of cases and primarily occur in patients aged 4–14 years. They are typically located on the buccal aspect of vital, unerupted, or partially erupted first or second permanent mandibular molars, usually presenting unilaterally and asymptomatically, though bilateral cases have been reported [3]. When symptomatic, they may cause localized discomfort and purulent exudate [4].

The etiology of BBC remains unclear, with several competing theories proposed. Kanno et al. [5] suggested that they may arise from reduced enamel epithelium and epithelial rests of Malassez, structures involved in paradental cyst formation. In contrast, Ackerman et al. [6] argued that if Malassez remnants were responsible, the lesions would occur uniformly around the root surface, which is not observed. Other authors have proposed alternative origins involving the sulcular epithelium, epithelial remnants of the dental lamina, or the junctional epithelium, reflecting the multifactorial nature of this condition [7].

The diagnosis of BBCs is based on the correlation of clinical, radiographic, surgical, and histological findings. Clinically, they present in early stages as a firm, palpable swelling in the buccal vestibule adjacent to a mandibular molar, often associated with delayed eruption and marked buccal inclination of the crown, resulting in lingual cusp prominence over the buccal cusps, while pulp vitality is generally preserved [8].

Radiologically, the lesion manifests as a radiolucent U‐shaped structure with well‐defined borders, situated relative to the vestibular surface of the roots. Its dimensions and extent vary. Both the periodontal ligament space and the lamina dura are preserved [9].

The aim of this article is to present a clinical case corresponding to a diagnosis of a BBC and its treatment, as well as to propose a novel management approach that contrasts with those reported in recent studies on the subject. This case report was prepared and reported in accordance with the CARE (CAse REport) guidelines, and the completed CARE checklist is provided as Supporting Information (Available here).

## 2. Case Report

The patient, aged 9 years, presented at the dental clinic for a periodic examination. The patient exhibited no pathologies or allergies, nor did he take any medication relevant to the case (ASA 1).

**Figure 1:**
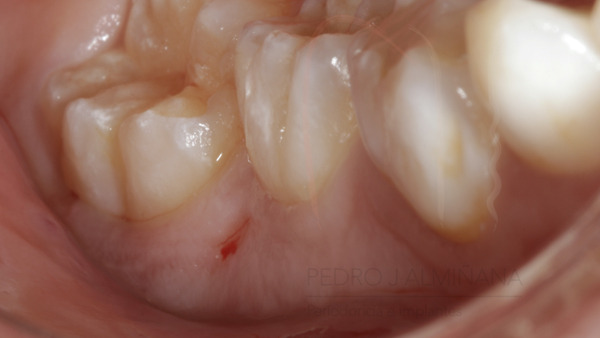
Intraoral photos. Clinical view of the buccal area of the fourth quadrant.

A thorough intraoral examination revealed mixed dentition, with fully erupted upper and lower incisors. The presence of the first permanent molars was noted, and the position of Molar 4.6 in infraocclusion, with the crown inclined towards the vestibular region, was particularly noteworthy (Figure 1). Upon palpation, a hard, nonpainful swelling was detected in the vestibular area of the molar.

No alterations were observed during the extraoral examination. The patient did not report pain or sensitivity.

**Figure 2:**
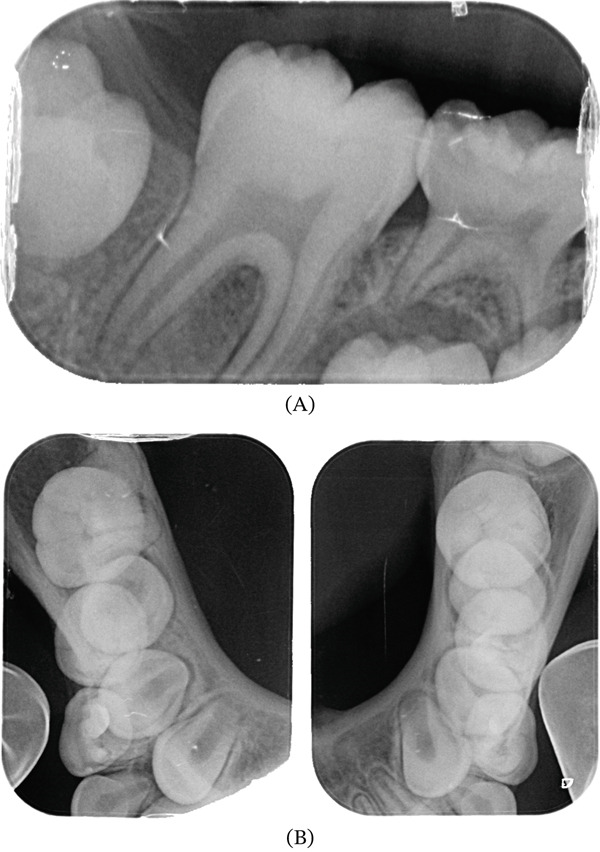
Radiography. (A) Periapical projection; and (B) occlusal projection.

**Figure 3:**
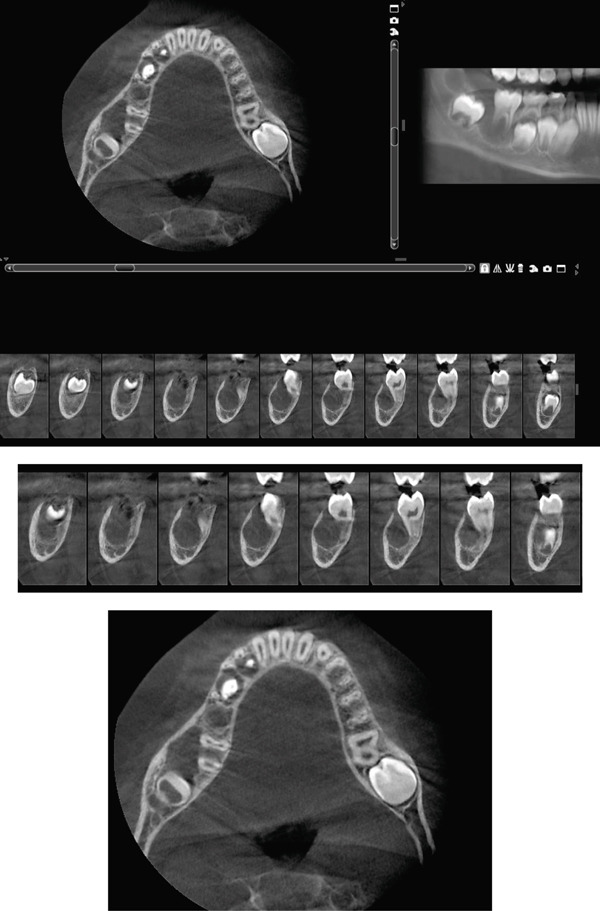
Radiography. CBCT, cone beam computed tomography.

The radiographic examination included an initial periapical projection, which revealed a possible loss of bone trabeculation in the furcation area when compared with the contralateral molar. This finding prompted the decision to proceed with an occlusal projection, revealing a buccal‐distal radiolucent area at the level of 4.6, which was also absent on the contralateral side (Figure 2). Given the suspicion of an inflammatory cyst of the bifurcation, a CBCT (cone beam computed tomography) was requested, which confirmed the location, extension, characteristics, and necessity for surgical treatment, thereby facilitating planning (Figure 3).

Given the substantial dimensions of the cyst, a minimally invasive procedure was deemed the optimal treatment modality. A lateral window osteotomy, analogous to the technique employed in sinus lift procedures, was performed to ensure complete excision of the lesion. The dimensions of this window were recorded as 4 mm in width and 3 mm in height.

The patient was administered moderate sedation with nitrous oxide and analgesia with both block and infiltration anesthesia to ensure optimal comfort during the procedure.

### 2.1. Surgical Procedure

A full thickness flap was executed utilizing an intrasulcular incision, complemented by two releasing incisions at the distalmost point of 4.6 and the mesial point of 8.4. Upon elevation of the mucoperiosteal flap, both the expansion of the vestibular wall and a communication to the coronal osseous crest were noted. However, the communication was too narrow to permit the removal of the lesion through this space. Given the dimensions of the cyst, it was hypothesized that an osteotomy with a lateral window would facilitate access and complete curettage of the lesion, preserving the height of the wall on which the flap would be repositioned. The 4 × 3‐mm micro‐osteotomy allowed access to the most apical tissues, enabling the detachment of the cyst without compromising the integrity of the capsule.

The cyst walls were resected, and the cavity was curetted from the lateral window and from the communication of the bony ridge with Tooth 4.6, using a Quincey tunneling knife (FQUINCEY2X Hu‐Friedy) and serrated Lucas spoon curettes. The excision of the cyst was deemed complete, and it was sent for histological analysis.

Due to the size of the lesion and its direct communication, a resorbable bovine pericardium collagen membrane was placed over the window (Botiss Jason 15 × 20 mm) following the principles of guided tissue regeneration (GTR) [10]. Finally, coronal repositioning of the flap was performed using 5‐0 polyglycolic acid suture (Anclasorb 3/8 DS 16).

**Figure 4:**
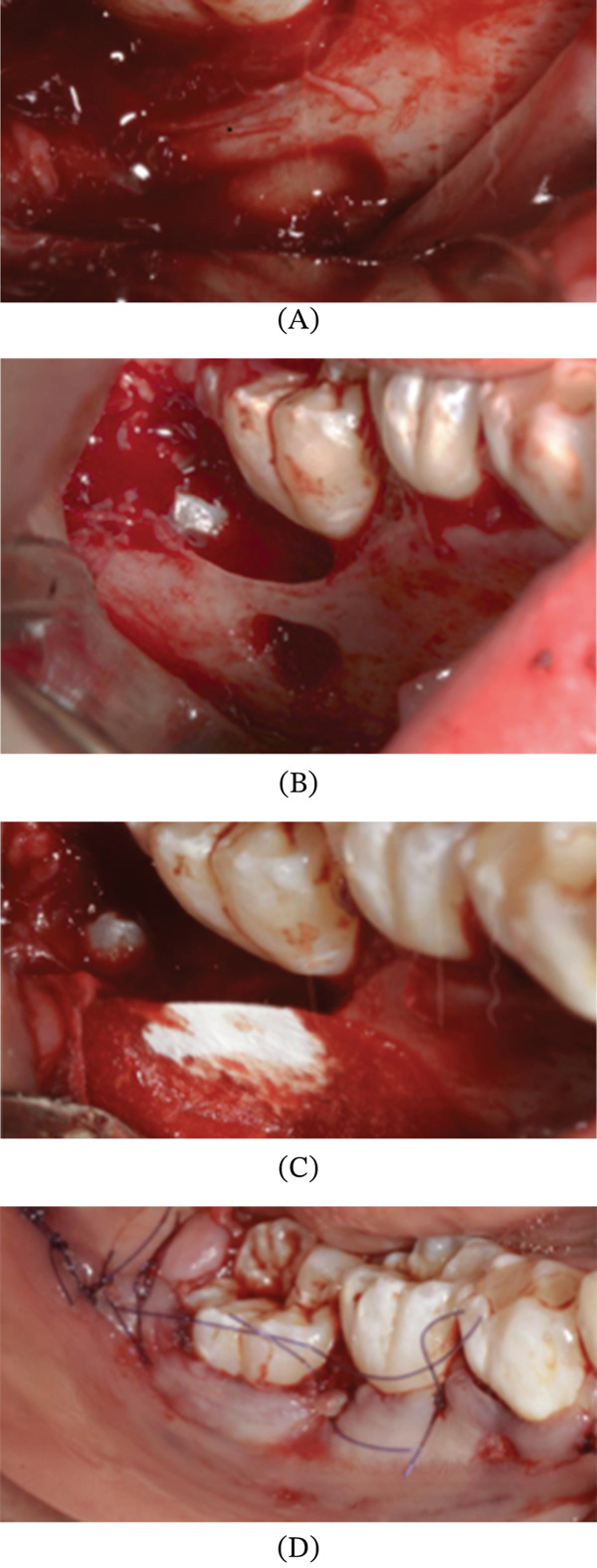
Surgical procedure: (A) micro‐osteotomy showing the bone window in the cortical bone and cyst′s soft tissue wall; (B) resection of the cyst with the bony cortex intact; (C) placement of the GTR collagen membrane; and (D) flap suture.

Postoperative guidelines were provided, which include the administration of amoxicillin 500 mg, ibuprofen 400 mg, and a 0.12% chlorhexidine mouthwash (Perio Aid). Sutures were removed 7 days after surgery (Figure 4).

The histological analysis of the extracted samples revealed the presence of an inflammatory cyst, with no signs of malignancy. Although histology is not considered a definitive diagnostic tool due to its uniformity across all inflammatory cysts, its analysis is instrumental in comprehensive documentation and diagnosis.

## 3. Results

**Figure 5:**
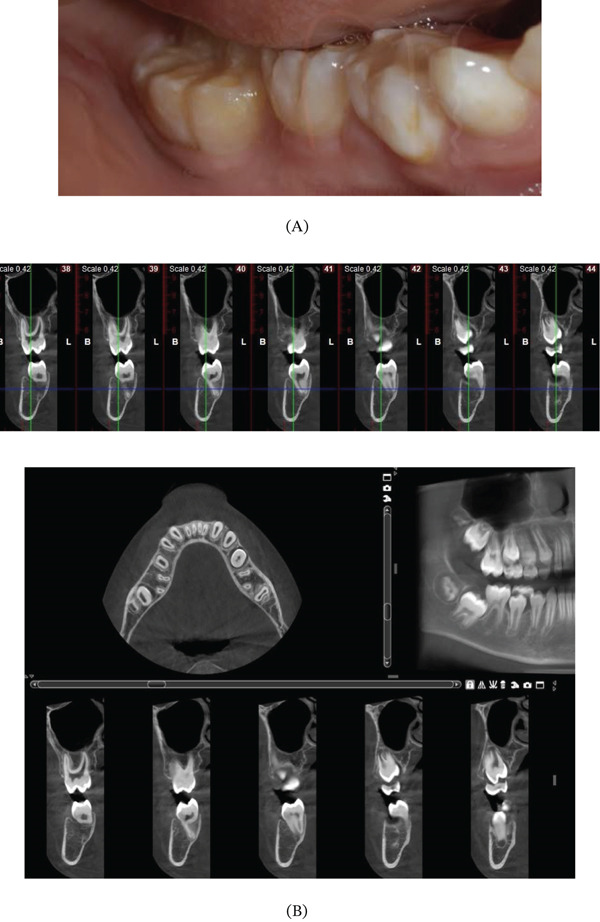
Final photos of the follow‐up: (A) Clinical view at 3 months; (B) at 1 year, a CBCT of the area confirms complete resolution.

At the 3‐month follow‐up, swelling was reduced, and the patient remained asymptomatic; by 6 months, the enlargement had almost resolved with noticeable improvement in tooth position. Radiographs at this stage showed increased bone trabeculation in the distal area of Tooth 4.6. One year posttreatment, a CBCT performed for orthodontic purposes confirmed complete osseodensification of the lesion and full recovery of the vestibular cortex (Figure 5).

## 4. Discussion

BBCs predominantly affect the mandibular first or second molars, with rare maxillary occurrences requiring differential diagnosis from keratocysts and lateral periodontal cysts [11].

Vitality of the involved molar is a key diagnostic criterion, as the cyst characteristically develops alongside a vital tooth, distinguishing it from lesions linked to pulpal pathology. The lesion is positioned laterally to the tooth, unlike dentigerous cysts that envelop the crown, helping differentiate it radiographically and clinically [6].

Radiographically, BBCs show maintained periodontal ligament space and lamina dura, with a distinct U‐shaped radiolucency in the furcation area and bone loss localized there; occlusal radiographs and CBCT are essential to define the lesion′s extent and aid diagnosis [12–14].

Early symptoms are often absent, so misdiagnosis as infection can delay appropriate treatment, which risks cyst expansion and tooth viability [2]. Literature reports treatment evolution from extraction and enucleation towards more conservative enucleation without extraction [4]. The use of bone substitutes as scaffolds limits true bone regeneration [15], providing radiographic fill but not histological restoration [16, 17].

This case contributes a novel surgical approach utilizing a precise 4 × 3‐mm micro‐osteotomy permitting enucleation of the cyst in one piece, combined with a bovine pericardial collagen membrane to promote GTR [18]. This membrane excludes epithelial cell invasion, fostering osteogenic cell repopulation and complete bone regeneration without bone substitutes [16, 17].

Twelve‐month CBCT follow‐up demonstrated full osseous defect regeneration and cortical restoration. Recent studies in related fields show membranes aid cortical bone integrity more than the absence of membranes does [19–22]. For extensive lesions or growing tissues, this minimally invasive, membrane‐assisted technique optimizes tissue regeneration and preserves anatomical structures, representing a technically advanced, conservative treatment alternative for BBCs.

## 5. Conclusions

The presence of a bulge in the vestibular area of a lower permanent molar, accompanied or not by infection, without an identifiable underlying cause, in conjunction with the patient′s age, the location and inclination of the molar, as well as radiological images, should prompt consideration of a MBBC.

The complementary explorations for diagnosis are the periapical and occlusal radiography, followed by a CBCT that will allow locating, sizing, and choosing the most appropriate therapeutic option.

A micro‐osteotomy and GTR are suggested as surgical techniques to fully regenerate tissue lost due to the cyst. These techniques are minimally invasive and do not require the use of any type of bone biomaterial.

**Nomenclature**
PC: paradental cyst
WHO: World Health Organization
GTR: guided tissue regeneration
CBCT: cone beam computed tomography
MBBC: mandibular buccal bifurcation cyst
ASA: American Society of Anesthesiologists

## Author Contributions

C‐P.M., A‐P.P., and B‐C.M. contributed to the conceptualization of the study. C‐R.M.D. and V‐R.M.A. performed the investigation. C‐P.M. provided supervision, help, and advice throughout the study. All authors contributed to the writing, review, and editing of the manuscript.

## Funding

No funding was received for this manuscript.

## Disclosure

All authors read and approved the final manuscript.

## Ethics Statement

Due to local regulations of Lluis Alcanyís Foundation Dental Clinic of the University of Valencia (Health Center 5452), no ethical approval is required for this retrospective case report. An informed consent for treatment and scientific diffusion of the case was signed by the parents and the patient, respectively.

Moreover, a second consent to publish “Patient Consent Form by Wiley” was obtained retrospectively from the patient during the preparation of the scientific article.

## Conflicts of Interest

The authors declare no conflicts of interest.

## Supporting information


**Supporting Information** Additional supporting information can be found online in the Supporting Information section. CARE checklist for case report.

## Data Availability

Data are available within the article and supporting information.
